# Persistent organic pollutants as risk factors for type 2 diabetes

**DOI:** 10.1186/s13098-015-0031-6

**Published:** 2015-04-30

**Authors:** Elvis Ndonwi Ngwa, Andre-Pascal Kengne, Barbara Tiedeu-Atogho, Edith-Pascale Mofo-Mato, Eugene Sobngwi

**Affiliations:** Laboratory of Molecular Medicine and Metabolism, Biotechnology Centre Nkolbisson, Biotechnology Centre Nkolbisson, Yaounde, Cameroon; Non-Communicable Diseases Research Unit, South African Medical Research Council, Cape Town, South Africa; Department of Medicine, University of Cape Town, Cape Town, South Africa; Department of Internal Medicine and Specialties, Faculty of Medicine and Biomedical Sciences, University of Yaoundé 1, Yaoundé, Cameroon; National Obesity Center, Yaoundé Central Hospital and Faculty of Medicine and Biomedical Sciences, University of Yaoundé 1, P.O. Box 7535, Yaoundé, Cameroon

**Keywords:** Type 2 diabetes, Persistent organic pollutants, In utero exposure, Insulin secretion, Insulin resistance

## Abstract

Type 2 diabetes mellitus (T2DM) is a major and fast growing public health problem. Although obesity is considered to be the main driver of the pandemic of T2DM, a possible contribution of some environmental contaminants, of which persistent organic pollutants (POPs) form a particular class, has been suggested. POPs are organic compounds that are resistant to environmental degradation through chemical, biological, and photolytic processes which enable them to persist in the environment, to be capable of long-range transport, bio accumulate in human and animal tissue, bio accumulate in food chains, and to have potential significant impacts on human health and the environment. Several epidemiological studies have reported an association between persistent organic pollutants and diabetes risk. These findings have been replicated in experimental studies both in human (in-vitro) and animals (in-vivo and in-vitro), and patho-physiological derangements through which these pollutants exercise their harmful effect on diabetes risk postulated. This review summarizes available studies, emphasises on limitations so as to enable subsequent studies to be centralized on possible pathways and bring out clearly the role of POPs on diabetes risk.

## Introduction

Type 2 diabetes (T2DM), the most common form of diabetes mellitus, is a complex condition resulting from the interaction between environmental and genetic factors. The global population of adults with diabetes was estimated to be 382 million (8 · 3%) in 2013 of which 175 million of cases are currently undiagnosed and this figure is predicted to rise to around 592 million (10.1%) by 2035 (International Diabetes Federation [1]).The majority of people with diabetes are aged between 40 and 59 years, and 80% of them live in low- and middle-income countries (IDF, International Diabetes Federation [1]). Higher-than-optimal weight resulting from the combined effects of unhealthy diets and decreased physical activity is a major driver of the ongoing pandemic of T2DM. Mounting evidences have also suggested the contribution of some environmental contaminants to the observed growing T2DM.

Persistent organic pollutants (POPs), one of the classes of environmental contaminants are a variety of chemicals which are either man-made or accidentally produced in industrial processes. The groups of compounds that make up POPs are also classed as PBTs (Persistent, Bio-accumulative and Toxic) or TOMPs (Toxic Organic Micro Pollutants). POPs are organic compounds that are resistant to environmental degradation through chemical, biological, and photolytic processes which enable them to persist in the environment, to be capable of long-range transport, bio accumulate in human and animal tissue, bio accumulate in food chains, and to have potential significant impacts on human health and the environment. POPs are generally classified into five main types which include polychloro-dibenzopara dioxins (PCDD) and polychloro-dibenzo furans (PCBF), poly-chlorinated biphenyls (PCBs), organo-chlorine (OC) pesticides, and poly-brominated flame retardants. The first three are also known as organo-chlorine compounds. Organo-chlorine compounds were banned in the late 1970s because of their toxicity. Lipid solubility, persistence in the environment and bioaccumulation potential in tissues through the food chain made these compounds to be very toxic. The bioaccumulation potential is due to the halogenated structure of the POPs. Human exposure to POPs occurs primarily through the consumption of animal fats like fatty fish, meat and dairy products [2]. There are geographic and socio-economic variations in type and route of exposure. Low and middle income countries population are at higher risk of exposure to POPs, either acute to high concentrations or chronic as long-term exposure to lower concentrations through diet, agriculture, occupation, and accidents especially for substances restricted or banned in developed countries. One of the POPs that is highly used in low and medium countries is dichloro-diphenyltrichloro-ethane (DDT)

DDT is a colourless, crystalline, tasteless and almost odourless organo-chloride which was initially produced and used to control malaria and typhus. It was used during the Second World War to control malaria and typhus among civilians and troops. After the war, DDT was made available for use as an agricultural insecticide. With the discovery of toxicological activity of DDT, its agricultural use was banned in the United States in 1972. By 1991, total ban on the use of DDT, including in disease control was implemented in about 26 countries. Due to the effectiveness of DDT against malaria and the burden of malaria especially in low and medium income countries, DDT even though banned is still used till date. Despite the possible role of DDT in the control of pest, its use should be avoided due to its negative effect on human health. It is classified as moderately toxic by the United States National Toxicology Program. Moreover, a number of studies from the US, Canada and Sweden have found that the prevalence of diabetes increases with serum DDT level. Several recent studies demonstrate a link between in-utero exposure to DDT and its metabolites and developmental neurotoxicity in humans. DDT has also been shown to induce neurological problems such as Parkinson’s and Alzheimer’s disease.

A review of the evidence on the association between organo-chlorine compounds and diabetes was published in 2005 [3]. However since then, new evidence have emerged to further substantiate such an association and putative mechanisms are increasingly tested, justifying an updated appraisal of existing evidences. Novel reviews have been published on the association between POPs and diabetes but limited to epidemiological studies [4,5]. Accordingly we reviewed the evidence from epidemiological and experimental studies on the association between exposures to persistent organic pollutants and type 2 diabetes risks. Additionally, we discussed the possible metabolic derangements and molecular mechanisms underlying such an association. We also explored evidence that would support the potential for exposure to POPs during foetal life to contribute to increased risk at adult age.

## Methods

The main databases used for the search of articles included in this study were PUBMED and HINARI. PUBMED search used the following key words: Type 2 diabetes + POPs, POPs + insulin resistance, POPs + insulin secretion, review + POPs + Diabetes, POPs + in-utero + exposure + diabetes. References from articles relating POPs to type 2 diabetes were used to search for the original articles through HINARI. Our inclusion criteria for epidemiological analyses comprised any cross sectional and prospective study with type 2 diabetes as the outcome published after 2005. For experimental studies, all available data on POPs and diabetes related complications were included. Studies on in-utero exposure to POPs and diabetes were obtained through the Google search engine to complement those from PUBMED.

### Evidence from epidemiological studies

#### Evidence prior to 2005

Evidence from epidemiological studies published before 2005 has been summarised previously by Arisawa et al. in 2005 who included 6 studies confirming an association between exposure to persistent organic pollutants and the risk of type 2 diabetes. These studies of which one was longitudinal and 5 cross sectional were published between 1997 and 2001. The main POPs included were PCDD, polychlorinated dibenzofuranes (PCDF) and PCBs, and exposure was mostly accidental in which individuals used substances containing POPs at work. They confirmed the association between POPs and type 2 diabetes but could not determine a causal relationship in the absence of experimental evidence [3].

#### Post 2005 epidemiological studies

Since the review by Arisawa et al. several other epidemiological studies have focused on the association between exposure to persistent organic pollutants and the risk of developing type 2 diabetes, using different designs. The characteristic of these studies are summarised in Tables 1 and 2.

**Table 1:** **Cross sectional studies of the association of POP with diabetes risk**

**Reference**	**Location**	**Population/** **setting**	**Age** ** (years)**	**Diagnostic criteria of diabetes**	**Confounders**	**Total population**	**Diabetic population**	**Proportion of men %**	**Compounds measured**	**Compounds with statistical significant difference**			
										**PCBs**	**Dioxins**	**OC pesticides**	**others**
[20]	Sweden	Fishermen and their wives from the Swedish east coast	66.5	Self-reported physician-diagnosed diabetes	Age, sex, BMI	380	22	51.6	PCB-153,p,p ′ -DDE	PCB-153 OR = 1.16, 95% CI (1.03-1.32)		p,p'-DDE OR = 1.05, 95% CI 1.01- 1.09).	
[8]	USA	NHANES (1999 – 2002)	≥20	FPG ≥ 126 mg/dl, Non-fasting glucose ≥200 mg/dl, Self-reported physician-diagnosed diabetes	Age, sex, BMI, race/ethnicity, poverty income ratio, WC	2,016	217	NA	PCB-153, HpCDD, OCDD, oxychlordane, DDE, trans-nonachlor	PCB-153 OR = 6.8, 95% CI (3.0–15.5)	HpCDD OR = 2.7, 95% CI (1.3–5.5)	Oxychlordane OR = 6.5 (2.0–21.4) DDE OR = 4.3, 95% CI (1.8–10.2);trans- nonachlor OR = 11.8, 95%CI (4.4–31.3)	
[9]	USA	Mohawk adults (one adult/ household)	57.5	FPG >125 mg/dl, Taking prescribed glucose-lowering medication	Age, sex, BMI, serum lipid levels, smoking history	352	71	38.1	Total PCB, PCB-153, **PCB-74**, DDE, HCB, mirex ,	Total PCB, OR = 3.9,95% CI (1.5–10.6). PCB-153 OR = 3.2, 95% CI (1.3–8.2) PCB-74 OR = 4.9, 95% CI (1.7–13.7)		DDE, OR = 6.4, 95%CI (2.2–18.4) HCB OR = 6.2, 95%CI (2.3–16.9)	
[21]	USA	NHANES (1982– 1984), Hispanic Americans	47	Self-reported physician diagnosed diabetes	Age, sex, BMI, place of birth, education, poverty index	1,303	89	40.5	**p,p ′ -DDT**, p,p ′ -DDE, dieldrin, oxychlordane, β -HCH, HCB, trans-nonachlor			p,p ′ -DDT (OR = 2.3,95% CI(1.1–5.0) , p,p ′ -DDE, OR = 2.63, 95% CI (1.2–5.8) β -HCH, OR = 2.7, 95% CI (0.9–8.2), oxy chlordane OR = 3.3, 95% CI (1.2–8.7) trans nonachlor OR = 3.4, 95% CI (1.4–7.9)	
Lee et al. [61]	USA	NHANES (1999 – 2002)	≥20	FPG ≥ 126 mg/dl, non-fasting glucose ≥ 200 mg/dl, Self-reported physician-diagnosed diabetes	Age, sex, BMI, race/ethnicity, poverty income ratio, WC	1,721	179	NA	PCDDs (3), PCDFs (3), dioxin-like PCBs (4), nondioxin-like PCBs (5),OC pesticides (4)	Dioxin-like PCBs OR = 15.7, 95% CI (3.4–71.2)		Oxychlordane, OR = 4.6, 95% CI (2.0–10.4),trans nonachlor, β –HCH Or = 7.0, 95% CI (2.7–18.1); DDT OR = 2.9, 95% CI (1.5–5.6),	
[23]	Sweden	Swedish fishermen’s wives	44	Self-reported diabetes	Age and BMI	544 women	16	0.0	CB-153 and p,p-DDE			CB-153; OR = 1.6, 95% CI (1. 0–2. 7) and p,p-DDE OR = 1.3, 95% CI (1. 1–1.6)	
[7]	USA	National Health and Nutrition Examination Survey 2003–2004	69	Fasting plasma glucose ≥126 mg/dl non-fasting plasma glucose ≥200 mg/dl , taking insulin or an oral agent.	Age, sex, race, poverty income ratio, and BMI	1,367	156	47.3	PBB-153 PBDE-28 PBDE-47 PBDE-99 PBDE-100 PBDE-153				PBB-153 OR = 1.9, 95% CI (0.9–4.0)
[26]	Japan	Survey (2002 – 2006)	44	Self-reported physician diagnosed diabetes, non-diagnosed participants with plasma HbA 1c >6.1%	age, gender, log (BMI), smoking habit, regional block, residential area, and survey year	1374	65	45.6	7 PCDDs, 10 PCDFs, 12 dioxin-like PCBs	12 dioxin-like PCBs OR = 6.82, 95% CI (2.59–20.1)	7 PCDDs,10 PCDFs OR = 2.21, 95% CI (1.02–5.04),		
[30]	Taiwan	Yu Cheng cohort (1993 – 2003)	55.5	Self-reported physician-diagnosed diabetes	age, BMI, cigarette smoking, and alcohol intake	748	144	41.0	Total PCB	Total PCB in women [OR 2.1,95% CI (1.1– 4.5)			
[62]	South Korea	Community-based health survey	55.6	FPG ≥ 126 mg/dL, taking antidiabetic medication	age, sex, BMI, alcohol consumption, and cigarette smoking.	80	40	52.5	β-hexachlorocyclohexane, HCB, heptachlor epoxide, p,p′-DDE, p,p′-DDD, p,p′-DDT, o,p′-DDT, oxychlordane, trans-nonachlor, and mirex			p,p′-DDE OR = 12.7, 95% CI (1.9–83.7),**p,p′-**DDT OR = 10.6, (1.3–84.9), o,p′-DDT OR = 12.3, 95% CI (1.3–113.2);oxychlordane, OR = 26.0, 95% CI (1.3–517.4); trans-nonachlor OR = 8.1, 95% CI (1.2–53.5) and heptachlor epoxide OR = 3.1, 95% CI (0.8–12.1)	
[19]	Slovakia	heavily polluted Slovakian district of Michalovce and two reference districts (Svidnik and Stropkov)	48	FPG >7.0 mmol/l (all participants) and 2 h glucose >11.1 mmol/l (OGTT, 60% of participants)	Age, sex, BMI	2,047	296	40.8	PCBs (15), p,p ′ -DDE, p,p ′ -DDT, HCB, β -HCH	Total PCBs OR = 2.74,95% CI (1.92 – 3.90)		p,p ′ -DDE, OR = 1.86,95% CI (1.17 – 2.95);p,p ′ -DDT OR = 2.48,95% CI (1.77–3.48)	
[24]	Finland	Helsinki Birth Cohort Study representing a general adult urban Finnish population	63.5	fasting plasma glucose ≥7.0 mmol/L or 2-h plasma glucose ≥11.1 mmol/L or on anti-diabetic medication.	Sex, age, WC and mean arterial pressure	1,988	308	46.3	oxy-chlordane, trans nonachlor, p,p’ -DDE, PCB 153, BDE 47, and BDE153	PCB 153 OR = 1.64 (0.92 – 2.93) P = 0.050		Oxy chlordane OR = 2.08, 95% CI (1.18 – 3.69); trans nonachlor OR = 2.24, 95% CI (1.25 – 4.03); DDE OR = 1.75, 95% CI (0.96 – 3.19)	
[25]	Japan	Participants in the Saku Control Obesity Program.	52	HbA1c level ≥ 6.9% ,taking medication for diabetes, fasting plasma glucose ≥ 126 mg/dL, or a history of doctor-diagnosed diabetes	Sex, age, BMI and total lipids.	117	32	50.4	PCB 74, 99, 118, 138, 146, 153, 156, 163/164, 170, 180 and 182/187	PCB 146, OR = 2.46, 95% CI (1.09-5.59) PCB 180 OR = 1.39, 95% CI (1.10-1.76)			
[34]	Denmark	The Gentofte population and Odense population	52.2	FPG ≥ 7.0 mmol/liter on two occasions, absence of anti-glutamic acid decarboxylase 65 antibodies, and no need for insulin therapy	Age, sex, and percent body fat, study site	148	31	64.3	HCB, HCH, p’p’-DDE, op’-DDE, p’p’-DDT, PCB-105, 118 and 156, PCB-101, 138, 153 and 180	PCB-105 OR = 3.8, 95% CI (3.0–4.9) PCB 118 OR = 14.2, 95% CI (10.2–21.0)		organochlorine HCB, OR = 28.0, 95% CI (20.0–39.7);p’,p’-DDE OR = 139.4,95% CI (73.6–288.4)	
[63]	Spain	Participants of the Catalan Health Interview Survey (CHIS)	46	FPG ≥ 126 mg/dL, reported physician diagnosed diabetes and current use of insulin or antidiabetic medication	age, sex, and BMI,total cholesterol and triglycerides	886	143	42.9	op-‘-DDT, p,p ′ -DDT, o,p′ -DDE, p,p ′ -DDE, o,p′ -DDD, p,p ′ -DDD; PCB28, 52, 101, 118, 138, 153, and 180, P**eCB**, HCB, α –HCH, β -HCH, γ -HCH, and δ -HCH	Non dioxin like PCBs (PCB28, 52, 101, 118, 138, 153, and 180,) OR = 1.8, 95% CI (1.2 − 2.7)		op-‘-DDT, o,p ′ -DDE, o,p ′ -DDD, p,p ′ -DDD; HCB, α –HCH,, γ -HCH, and δ –HCH OR = 1.8, 95% CI (1.0 − 3.2)	
[6]	USA	Employed at the La Salle Electrical Utilities Company in illinios and local residents not previously employed	57	self-reported diagnosed diabetes, and/or fasting glucose ≥ 126 mg/dL	age, BMI, alcohol use, smoking, high blood pressure, total lipids, medication use	63	7	100	6 dioxin-like PCBs and 33 non dioxin like PCBs	All PCBs OR = 3.0, 95% CI (1.3-7.2)			
[18]	USA	Anniston, Alabama general population	55.5	fasting glucose values > 125 mg/dL, self-reported or physician diagnosed diabetes	Age, sex, race/ ethnicity, or BMI	774	207	30.0	35 PCB ortho-substituted congeners, DDE	All PCBs 2.78,95% CI(1.00-7.73)		DDE OR = 1.22, 95% CI(0.85-1.46)	
[64]	Spain	San Cecilio University Hospital in thecity of Granada and Santa Ana Hospital in the town of Motril	55	self-reported information and clinical records showing a fasting glucose level Z 126 mg/dL in the routine analyses	Adipose tissue origin , sex, age, and BMI	386	34	51.0	PCB-138, PCB-153, PCB-180, HCB, b-HCH, p,p’-DDE,			p,p‘-DDE OR = 4.4, 95% CI (1.0–21.0)	
[65]	Canada	Wapekeka and Kasabonika two of the five Shibogama communities in northern Ontario	44.5	resting plasma glucose >7.0 mmol/L and/or a post-prandial level (2 hours after glucose ingestion) > 11.0 mmol/L	Age, BMI and smoking status	72	26	43.1	Aroclor 1260, PCB28, 52, 99, 101, 105, 118, 128, 138,153, 156, 163, 170, 180,183,187,aldrin, − αchlordane, β-chlordane, − β-HCH, cis-nonachlor, trans-nonachlor, DDE, DDT, HCB,mirex, oxychlordane, PBB153, PBDE47, 99, 100,153, Parlar26, and Parlar50.	PCB28, 52, 99, 101, 118, 128, 138, 153, 156, 163, 170, 180, 183,187,		Trans nonachlor, DDE, oxychlordane, all p < 0.05	
[66]	South Korea	NA	NA	FPG ≥ 126 mg/dL, reported history of physician-diagnosed diabetes	age , sex, alcohol consumption , cigarette smoking and BMI	50	25	48.0	14 OC pesticides, 22 PCB congeners			DDTs in VAT, OR = 9.0,95% CI(1.3–62.9)	
	South Korea	Community-based health survey	55.6	FPG ≥ 126 mg/dL, taking antidiabetic medication	age, sex, BMI, alcohol consumption, and cigarette smoking.	80	40	52.5	β-hexachlorocyclohexane, HCB, heptachlor epoxide, p,p′-DDE, p,p′-DDD, p,p′-DDT, o,p′-DDT, oxychlordane, trans-nonachlor, and mirex			p,p′-DDE OR = 12.7, 95% CI (1.9–83.7);**p,p′-**DDT OR = 10.6, 95% CI (1.3–84.9);o,p′-DDT OR = 12.3, 95% CI (1.3–113.2);oxychlordane, OR = 26.0, 95% CI (1.3–517.4); trans-nonachlor OR = 8.1, 95% CI (1.2–53.5) and heptachlor epoxide OR = 3.1, 95% CI (0.8–12.1)	

2,2,4,4,5,5-hexabromophenyl (PBB-153), heptachlorodibenzo-p-dioxin (HpCDD), octachlorodibenzo-p-dioxin (OCDD), dichlorodiphenyldichloroethylene (DDE), 2,2′-bis(4-chlorophenyl)-1,1-dichloroethylene (p,p′-DDE), hexachlorobenzene (HCB), hexa-chlorocyclohexane (HCH), polychlorinated dibenzo-p-dioxins (PCDDs), polychlorinated dibenzofurans (PCDFs), organochlorine (OC), 2,2',4,4'-tetrabromodiphenyl ether (BDE 47), 2,2',4,4',5,5'–hexabromodiphenyl ether (BDE 153), 2,2,4,4,5,5-hexabromophenyl (PBB-153), 2,4,4-tribromodiphenyl ether (PBDE-28), 2,2,4,4 tetrabromodiphenyl ether (PBDE-47), 2,2,4,4 ,5-pentabromodiphenylether (PBDE-99), 2,2,4,4,6-pentabromodiphenyl ether (PBDE-100), 2,2,4,4,5,5-hexabromodiphenyl ether (PBDE-153), 2,2 ′ -bis(4-chlorophenyl)-1,1,1-trichloro-ethane (p,p ′ -DDT), 2,2′-bis(4-chlorophenyl)-1,1-dichloroethylene (p,p′-DDE), di-chlorodiphenyl-dichloro-ethane (o,p′-DDD ), pentachlorobenzene (PeCB), body mass index (BMI), confidence interval (CI), fasting plasma glucose (FPG), oral glucose tolerance test (OGTT), odd ratio (OR),waist circumference (WC).

**Table 2:** **Longitudinal studies of the association of POP with diabetes risk**

**Reference**	**Location**	**Population/setting**	**Mean Age (year)**	**Diagnostic criteria of diabetes**	**Confounders**	**Number of participants**	**Number of diabetic participants**	**Proportion of men**	**Duration of follow-up (years)**	**Compounds measured**	**Compounds with statistical significant difference**			
											**PCBs**	**Dioxins**	**OC pesticides**	**Others**
[15]	USA	Michigan PBB cohort	≥ 20	Self-reported physician-diagnosed diabetes, anti-diabetic medication	Age, body mass index, smoking, and alcohol consumption	1384	180	49.7	25	PBB, PCB	PCB in women Incidence Density Ratio [IDR = 2.33; (95% CI 1.25– 4.34)]			
[17]	USA	Veterans of Operation Ranch Hand		medical diagnosis or 2-hour postprandial glucose ≥200 mg/dL	BMI, smoking history, family history of diabetes	3049	439 men	100	10	TCDD		TCDDRR = 1.32, p = 0.04		
[10]	Sweden	WHILA cohort (1995 – 2000)	54.5	Baseline OGTT	Age, calendar year, BMI, heredity, country of birth, education, smoking, alcohol intake, hormone Replacement therapy, physical activity	742	371 women	0.0	10	PCB-153, p,p ′ -DDE	CB-153 OR = 1.6 (95% 0.61-4.0)		p,p ′ -DDE OR = 5.5 (95% CI 1.2-25)	
[12]	USA	Cross-section of Great Lakes sport fish consumers (1992, follow-up 2004 – 2005)	50.5	Self-reported physician-diagnosed diabetes,	Age, BMI, sex, serum lipids, smoking, alcohol use, all fish meals in the last year	471	36	59.2	10	PCB 74, 99, 118, 146, 180, 194, 201, 206, 132/153, 138/163, 170/190, 182/187, 196/20, DDE			DDE RR = 2.01 (95% CI 1.20–3.66)	
[31]	USA	CARDIA cohort study (1985 – 1986; follow up: 1987 – 2006)	24	Use of glucose-lowering medications	Age, sex, BMI, race	180	90	38.4	18	OC pesticides (9), PCBs (35), PBDEs (1), PBB (1)	Highly chlorinated PCBs OR = 2.8 (95% CI1.1–7.0)		Trans Nonachlor OR = 4.8 (95% CI1.7–13.7)	PBB-153 OR = 3.0 (95% CI1.1–8.1)
[11]	Sweden	General population of Uppsala	70	fasting blood glucose ≥6.2 mmol/L or the use of insulin or oral hypoglycemic agents	sex, BMI, cigarette smoking, alcohol consumption, and total lipids	725	36	48.3	5	PCB74, 99,105, 118, 138, 153, 156, 157, 170, 180, 189, 194, 206, 209, p,p’- DDE, Trans –nonachlor, Hexa-chloro benzene, brominated diphenyl ether 47, dioxin	PCB74, 99,105, 118, 138, 153, 156, 157, 170, 180, 189, 194, 206, 209 OR = 7.5 (95% CI1.4 – 38.8)		DDE, Trans nonachlor and Hexa-chloro benzene, OR = 3.4 (95% CI1.0 – 11.7)	
[14]	USA	Nurses’ Health Study.	42.5	Self-reported diagnosed diabetes, and/or fasting glucose ≥ 126 mg/dL	Age, BMI, smoking status, alcohol intake, physical activity and family history of diabetes	1,095 women	48	0.0	18	PCBs, DDT, DDE and HCB			HCB OR = 3.59 (95% CI 1.49-8.64)	

2,2,4,4,5,5-hexachlorobiphenyl (CB-153), 2,2′-bis(4-chlorophenyl)-1,1-dichloroethylene (p,p′-DDE), polychlorinated biphenyls (PCBs), pentabromo-diphenylether (PBDEs), hexabromophenyl (PBB) tetrachlorodibenzo para dioxine (TCDD), di-chlorodiphenyl-trichloro-ethane (DDT), dichlorodiphenyldichloroethylene (DDE), hexachlorobenzene (HCB); body mass index (BMI), oral glucose tolerance test (OGTT).

### Evidence from cross-sectional and case–control studies

Since 2005, at least 20 cross-sectional studies conducted in about 12 countries have been published on the association of POPs with diabetes risk. The number of participants across studies ranged from 65 to 2047, the proportion of men from 30.0% to 64.3% and the proportion with diabetes from 5.8% to 27.4%. Even though with a diabetes prevalence of 11%, the number of diabetes cases was the least (2) in the study of [6]. The association between POPs and diabetes was based on comparing the mean value of POPs in diabetic and non diabetic individuals. Diagnosis of diabetes varied across studies and was based on FPG ≥126 mg/dl, non-fasting glucose ≥200 mg/dl, self-reported physician-diagnosed diabetes, taking prescribed glucose-lowering medication, non-diagnosed participants with plasma HbA1c >6.1%, 2-h plasma glucose ≥ 199.8 mg/dL, and HbA1c level ≥6.9%. Over 58 POPs compound were investigated in those studies with a minimum of two and a maximum of 36 per study. Measures of associations used to assess the effect of these compounds on diabetes risk across studies included differences in the prevalence of diabetes amongst groups, odd ratios which were often adjusted for common diabetes risk factors like age, sex, body mass index, race/ethnicity, alcohol use, smoking, high blood (BP) pressure or BP indices, lipids, medication use and waist circumference. Exposure was expressed as categorized variables ranging from two groups to quintiles with the group having low plasma POP constituting the control group. The odd ratio in the fifth quintile compared to the reference group ranged from 1.9 for PBDE-153 Lim et al. [7]) to 37.7 [8]. Stratification in tertiles gave an OR of 3.9 (95% confidence interval, 1.5–10.6) for total PCBs [9]. PCB-153,pp’-DDT, and pp’-DDE were associated with type 2 diabetes in all studies while non dioxin like PCBs, HCB, BDE-47, BDE-153 and HCH were associated with diabetes only in one study (Table 1).

### Evidence from longitudinal and time-series studies

Since 2005, at least 7 longitudinal studies were published from about 3 countries reporting on the association of POPs with diabetes risk. The number of participants across studies ranged from 180 to 3049. The proportion of men ranged from 38.4% to 59.2% and the proportion of participants with diabetes from 4.4% to 19.3%. Two studies were considered to be case control since the prevalence of diabetes was very high compared to that of the normal population. The prevalence was 50% in both studies [10,11]. In the study conducted by Rignell-hydbom et al., [10], the controls were randomly selected from the same cohort with the cases (Women's Health In the Lund Area cohort) and matched for age, calendar year, BMI and presence or absence of metabolic syndrome at baseline. The second study also included all participants from the same cohort whereby participants who developed diabetes during the follow-up were considered cases and those who were diabetes free were taken as controls. Diagnosis of diabetes varied across studies and was based on self-reported physician-diagnosed diabetes, baseline OGTT, taking glucose-lowering medications, fasting blood glucose ≥111.6 mg/dL (≥6.2 mmol/L) non-diagnosed participants with HbA1c >6.1% (or >6.3%), verified history of diabetes by medical diagnosis, 2-hour postprandial glucose ≥200 mg/dL, or fasting 5 glucose ≥126 mg/dL. Over 51 POPs compound were tested in those studies with a minimum of two and a maximum of 46 compounds per study. The duration of follow-up in studies varied between 5 and 25 years (mean = 13.2). Measures of associations used to assess the effect of these compounds on diabetes risk across studies included differences in the incidence of diabetes amongst groups, odd ratios, least squares geometric means, hazard ratios, incidence density ratios (IDRs), and relative risk which were often adjusted for common diabetes risk factors like age, body mass index, cigarette smoking, country of birth, education, alcohol intake, hormone, physical activity, race, lipids and family history of diabetes. Hexachloro benzene (HCB), 2,3,7,8-tetrachlorodibenzo-p-dioxin (TCDD) and 2,2′-bis(4-chlorophenyl)-1,1-dichloroethylene (pp’-DDE) were associated with the risk of type 2 diabetes while the association was found only in women when PCBs were investigated. pentabromodiphenylethers (PBDEs), 2,2',4,4'-tetrabromodiphenyl ether (BDE-47), dichloro-diphenyl-trichloro-ethane (DDT) and non-dioxin like PCB were associated with the risk of diabetes in none of the studies (Table 2).

In all the longitudinal studies included in this review, bio-monitoring was performed from baseline until the end of the study. This is shown by the fact that all participants included were diabetes free at baseline and only developed diabetes during the follow-up and participants with diabetes before baseline were excluded from the study. Bio-monitoring of POPs was carried out only in one of the seven studies because exposure was accidental. Exposure in the other studies was background and follow up was carried out to determine incident diabetes cases ([11,12]; Lee et al., [13,14]; Vasiliu et al., [15]; RignellHydbom et al., [10]). In the study conducted by RignellHydbon et al., [10] blood samples were stored for at least three years at −70°C before POPs analysis. Analysis of POPs was performed at the end of follow-up. Other studies analyzed POPs at enrolment with blood samples store at −20°C or −70°C (Lee et al., [16]; Michalek and Pavuk., [12,14,17]). Moreover, analysis of POPs was performed once throughout the follow-up in most of the studies ([11]; Lee et al., [14,16]; Vasiliu et al., [15]; RignellHydbon et al., [10]). Even though multiple analyses of POPs was carried out by [17], only one point measurement was used in the analysis since veterans who participated in at least one of the last five examinations were included in the study. Reverse causality can be a possible association between type 2 diabetes and POPs where individuals with diabetes are unable to metabolise POPs resulting to a higher concentration compared to non diabeteic individuals. This can be evaluated in studies with repeated measurements POPs [12].

### Limitations and strengths of the available epidemiological studies

Several epidemiological studies have evaluated the association between POPs and diabetes using accidental exposure to PCBs [6,18] and high zone exposure to organo-chlorine (OC) pollutants [19]. These exposure rates are higher than those observed in the general population. Therefore, results from these studies can only evaluate the health risk of persistent organic pollutants but cannot be generalised in the total population more often affected by chronic low concentration exposure. Moreover most of the studies with background exposure to polychlorinated biphenyls (PCBs) and organochlorine pesticides ([9,20,21]; Lee et al. [22-25]) had a cross sectional design. As such, a causal relationship cannot be established considering for instance the possibility that diabetic individuals may have a reduced capacity to excrete or metabolise pollutants leading to a high concentration of POPs in diabetic individuals compared to those without. In-depth investigation of the effect of chemicals on the processes leading to the development of diabetes is thus required. The small sample size and prevalence of diabetes in some studies reduced to unstable estimates of the association. Moreover, Michalek and Pavuk did not observe any difference in diabetes prevalence between the exposed and the unexposed groups [17]. The study was aimed at assessing the association between 2,3,7,8-tetrachlorodibenzo-p-dioxin (TCDD) and diabetes and TCDD and cancer in veterans involved in the spraying of Agent Orange and other TCDD contaminated herbicides in Vietnam from 1962 to 1971. Two groups were involved in the study; one responsible for the spraying of Agent Orange and a comparing group a cohort of other Air Force veterans who served in the Southeast Asia (SEA) region during the same period that the Ranch Hand unit was active but who did not spray herbicides. The test group was exposed to Agent Orange contaminated with TCDD during the Vietnam War. Despite the lack of association between the two groups, stratification by calendar period of service and days of spraying revealed a significant increase in the risk of diabetes among Ranch Hands who served during or before 1969 and who had at least 90 days of spraying [17]. This study suggested that the risk of type 2 diabetes might be associated with the duration of exposure which can better be evaluated in longitudinal studies

Longitudinal studies have been carried out to establish a causal relationship between type 2 diabetes and POPs (Vasiliu et al. [15], Rignell-Hydbom et al. [10-12], Lee et al., [14,16]) although exposure rates were higher than those observed in the general population. However, a study carried out among general inhabitants in Japan confirmed a high risk of diabetes in individuals with high level of Dioxin [26]. The most convincing evidence for a relationship between diabetes and persistent organic pollutants (POPs) in humans comes from accidental or occupational exposures to high levels of these compounds. This is documented by David O Carpenter in a review entitled "Environmental Contaminants as Risk Factors for Developing Diabetes". The studies carried out in individuals occupationally exposed to high level of this class of POPs showed an increase in the prevalence and incidence of diabetes compared to the general population. There was a significantly elevated deaths from diabetes among women exposed to dioxins at Seveso, Italy living in the zone with intermediate exposure (OR = 1.9, 95% CI: 1.1-3.2) [27]. Analysis of data reported from 36 international cohorts of workers in phenoxyacid herbicide and chloro-phenol plants, where dioxins were an un-intended by product reported a RR of 2.25 (95% CI: 0.53-9.50) for diabetes in these workers [28]. Moreover, 60% of workers in two US chemical plants had diabetes and workers with the highest extrapolated TCDD concentrations had a significantly increased mean serum glucose concentration [29]. All these studies substantiate the fact that high exposure to POPs from occupational and accidental setting increases the risk of type 2 diabetes.

Epidemiological associations between persistent organic pollutants and type 2 diabetes have been widely assessed in Europe, Asia and America. However, the intentional use has been restricted or banned in many developed countries. Studies on the association between these contaminants and diabetes in developing countries particularly Africa are lacking while the prevalence of diabetes is predicted to increase the highest in the region. Some studies [12,20,21,23,30] used only self-reported physician-diagnosed diabetes as a diagnostic criteria for diabetes. A false diagnosis will modify the prevalence or incidence rate and in turn dilute estimates of the association. Moreover, differences in the diagnosis criteria make it impossible to compare the magnitude of the association across studies.

### Metabolic derangements associated with exposure to POP

Insulin resistance and defects in insulin secretion have been investigated in in-vitro and in-vivo experimental studies, as the possible metabolic derangements linking exposure to POPs and diabetes occurrence. Insulin plays a fundamental role in the uptake of glucose by the adipose tissue, liver and muscles. Insulin exerts its action by binding to its receptor and initiating a cascade of reactions culminating with the translocation of the glucose transporter from the endoplasmic reticulum to the plasma membrane for glucose uptake. Competition between insulin and other ligands for the receptor as well as modulation of the expression of genes involved in the signaling cascade will lead to an insulin resistance state. The overproduction of insulin by the pancreatic beta cell to compensate for the insulin resistance state might lead to the exhaustion of the cells and future type 2 diabetes. POPs have been shown to affect these pathways both in human and animal studies.

### Human studies: effect of POPs on insulin secretion and insulin resistance

Few studies in human subjects have reported that POPs increases the risk of insulin resistance both in diabetic and non-diabetic individuals [13,22,31,32]. In a study carried out in non-diabetic individuals with background exposure to TCDD, it was observed that individuals with level of TCDD greater than 15 ppb had high fasting and pros-prandial insulin levels after a glucose load compared to individuals with low level TCDD, suggesting that high blood TCDD may cause insulin resistance [31]. Another study focusing on low dose exposure to organo-chlorine (OC) pesticides and PCBs observed a non monotonic dose response (U shape) association between POPs and insulin resistance in non-diabetic individuals [16]. Toxicity is assumed to be directly proportional to the dose of the chemical. However, some substances especially endocrine disruptors may cause biological response at doses well below those levels previously tested and determined to be safe. A U-shape curve is therefore obtained between exposure and outcome when low exposure corresponding to high biological effect is considered to be the control group. However other studies have not found an association between POPs and insulin resistance defined by HOMA-IR [6,33]. Some have suggested an effect of POPs on insulin secretion instead of insulin resistance [33].We found only one published study exists on the association between POPs and insulin secretion in human subjects. Jørgensen and collaborators observed a significant negative association between POPs and indices of insulin secretion: stimulated insulin and HOMA-B indicating that POPs may affect basal and stimulated insulin secretion [33]. Studies with accurate measurement of insulin secretion and insulin action are warranted. In fact, few investigations used clamp studies or other dynamic testing. Two studies have evaluated the association between POPs and insulin action using euglycemic hyper-insulinaemic clamp as a measure of insulin action. Færch et al. used two different clamp procedures. In one study group, a euglycemic-hyperinsulinemic clamp at 40 mU/m^2^.min for 4 h was performed after a 2-h basal period. In the second group, the 2-h basal period was followed by a 30-min IVGTT for assessment of first-phase insulin secretion [34]. Immediately after the IVGTT, a euglycemic-hyperinsulinemic clamp at 40 mU/m^2^.min for 2 h was performed. In their study, individuals with prediabetes and diabetes had higher serum concentrations of several POPs (HCB, p,p’-DDE, p,p’-DDT, PCB-118 and PCB-156) compared with normoglycemic individuals. Moreover, in the non-diabetic population, higher POPs levels were associated with elevated fasting plasma glucose concentrations as well as reduced glucose oxidation, elevated lipid oxidation, and elevated serum concentrations of free fatty acids. However, after exclusion of diabetic patients, there were no differences between the two study populations with regard to fasting plasma glucose, serum insulin, HOMA-IR, or HOMA-B [34]. Gauthier et al. in their study measure insulin sensitivity using the 3-hour hyperinsulinemic-euglycemic clamp as described by Defronzo [35]. Despite the fact that an association between POPs and insulin resistance was not evaluated in this study, it could be deduced from the results that insulin sensitivity characterized by 3-hour hyperinsulinemic-euglycemic clamp was inversely associated with the concentration of POPs. This is because insulin sensitivity was higher in metabolically healthy but obese 8 individuals who had a lower concentration of POPs compared to metabolically abnormal obese individuals with a higher concentration of POPs [36].

### Animal studies: effect of POPs on insulin secretion and insulin resistance

The effects of POPs on insulin secretion and insulin resistance have been evaluated in few animal studies. The first animal study to observe an effect of POPs on insulin resistance was carried out in Sprague–Dawley rats [37]. These rats were exposed for 28 days to lipopholic POPs through the consumption of high fat diet containing either refined or crude fish oil obtained from farmed Atlantic salmon. Insulin sensitivity was assessed in the soleus muscle and epididymal fat using the hyper-insulinemic-euglycemic clamp technique. Furthermore, cultured and differentiated 3 T3-L1 cells were used to assess insulin-stimulated glucose uptake in-vitro. There was a significant inhibition of insulin-dependent glucose uptake in skeletal muscle of animals fed on high fat diet containing POPs (in-vivo) as well as differentiated adipocytes treated with nano-molar concentrations of POP (in-vitro) [37]. Moreover, C57BL/6 J mice fed a very high-fat (VHF) diet containing farmed Atlantic salmon fillet (a source of POPs) for 8 weeks when compared with another group of very high fat diet without POPs or chow diet, had a significant high fasting and pros-prandial glucose level [38].There was also a reduced response to glucose clearance following insulin load relative to VHF- and Chow-fed mice all, features demonstrating a state of insulin resistance [38].Administration of PCB-77 or PCB-126 has also been shown to impair glucose homeostasis in lean mice and in obese mice [39].

### Molecular mechanisms linking persistent organic pollutants exposure with metabolic derangements

Amongst the different classes of persistent organic pollutants (POPs), 2,3,7,8-tetrachlo-rodibenzo-p-dioxin (TCDD) has been extensively studied and the possible mechanism of disruption of beta cell function reviewed [40]. One prominent symptom of acute toxicity from TCDD is a loss of adipose tissue and body weight, a phenomenon known as the wasting syndrome [41]. This syndrome is characterised by hypophagia, hyper-lipidaemia and hyper-triglyceridaemia suggesting that affected animals are unable to utilize the energy-rich nutritional compounds available in their blood. The main pathway of TCDD action is through the aryl hydrocarbon receptor. Binding of TCDD to aryl hydrocarbon receptor causes changes in translational and transcriptional mechanisms resulting in diminished GLUT expression (Enan et al., [42]). Inhibition of GLUT translocation from the cytoplasm to the plasma membrane as well as reduced glucose transporter availability limits the uptake of glucose which is a hallmark of insulin resistance. A decrease in the level of mRNA of the glucose transporter (GLUT) gene and protein levels as well as the glucose uptake was observed in TCDD treated P19 cells [43]. Moreover, treatment of 3 T3-L1 adipocytes with 10nM TCDD caused decreases in the expression levels of insulin 9 receptor beta (IRβ), insulin receptor substrate 1 (IRS1) and GLUT4 and insulin-stimulated glucose uptake activity, strongly suggesting that TCDD triggers dysfunction of the insulin signalling pathway in adipocytes [44]. Even low doses of TCDD (0.03 pg/kg) causes a significant reduction in the glucose transporting activity of guinea pig adipose tissue and pancreas [45]. Compared with TCDD, the effects of polychlorinated biphenyls (PCBs) on insulin secretion are less thoroughly studied. However, a more recent study investigated the in-vitro and in-vivo effects of coplanar PCBs on adipose expression of tumour necrosis factor α (TNF-α) and on glucose and insulin homeostasis in lean and obese mice [39]. In cultured adipocytes, PCB-77 promoted TNF-α expression through an AhR-dependent mechanism suggesting that PCBs could promote insulin resistance through adipose-specific increases in TNF-α [39].

Maintenance of blood glucose homeostasis by the endocrine system involves a series of complex gene-environment interactions between different tissues, including the liver, skeletal muscle, adipose tissue, brain and the pancreas. Altered glucose homeostasis leads to type 2 diabetes, which consists of defects in both insulin secretion and insulin action (insulin resistance) [46]. Since the beta cells of the pancreas are central to controlling glucose homeostasis, an endocrine disrupting chemical that can initiate, facilitate and/or accelerate the loss of beta cell function can play an important role in type 2 diabetes. Moreover, obesity the main predictor of type 2 diabetes is a complex endocrine-related disease caused by the interaction between genetic, behavioral, and environmental factors [47]. Therefore endocrine disruption could be the possible link between obesity and diabetes. Moreover, POPs have also been shown to possess endocrine disruption activities through interaction with estrogen receptors [48] and peroxisome proliferator receptor (PPAR) receptor [49]. Endocrine disruptors are chemicals that, at certain doses, alter the normal functioning of hormones and other signaling molecules in the body. The endocrine disrupting activities of these POPs include their effects on thyroid hormone. Thyroid hormone is essential for normal brain development, for the control of metabolism, and for many aspects of normal adult physiology. Therefore, changes in the functions of the thyroid gland or interference with the ability of thyroid hormones to exert their actions may produce effects on development, metabolism, or adult physiology. POPs interfere with the thyroid function by acting on different points of regulation of thyroid hormones synthesis, release, transport through the blood, metabolism of thyroid hormones, and thyroid hormones clearance [50]. The best example is PCB which can reduce circulating levels of thyroxine (T4) in animals (Bastomsky., [51,52]).

### In-utero exposure to POPs and diabetes risk

In-utero exposure to several factors including persistent organic pollutants (POPs) has been shown to increase the risk of many diseases. In pregnant women, POPs have been shown to cross the placenta [53]. The developmental origin of health postulates that adverse foetal exposure may lead to permanent foetal adaptations in structure, physiology and metabolism. These adaptations might be beneficial for short term foetal survival, but may lead to foetal growth retardation, cardiovascular and metabolic diseases in adulthood. The association between intrauterine exposure to persistent organic pollutants and birth weight has been highly investigated. However findings from these studies are inconsistent and the association has been reviewed by Govarts et al. [54]. In the meta-analysis consisting of European birth cohorts they observed that decrease in birth weight independently of gestational age was associated with increasing foetal PCB-153 concentrations. [54]. Across all cohorts birth weight declined by 150 g for each 1-μg/L increase of PCB-153 in cord serum. However, no association was observed with the DDT metabolite p,p´-DDE. Due to inconsistent results in previous studies, other studies (summarised in Table 3) have been carried out to examine the nature of the association. It was observed that higher concentrations of PBDEs in maternal serum during pregnancy were associated with lower birth weight in a population of low-income women living in California [55]. Moreover a 10-fold increase in concentrations of BDE-47,-99, and −100 was associated with an approximately 115-g decrease in birth weight. Another study found a significant reduction in birth weight and length of gestation associated with maternal CB-153 exposure among Inuits but not in the Kharkiv or War-saw cohorts [56]. A cohort study from Valencia revealed that prenatal exposure to OCs may exert adverse health effects on birth size, reducing the birth weight, length, and head circumference as shown by a significant decrease of 63 and 107 g in birth weight with each 10-fold increase in cord serum 4,4’-DDT and 4,4’-DDE concentrations, respectively [57]. A more recent study found a weak but significant positive association between background prenatal exposure to di-ortho PCBs and birth weight [58]. However, others studies showed that intrauterine exposure to some persistent organic pollutants was not associated with birth weight [59]. A large number of published studies have also reported negative associations between in-utero exposure to persistent organic pollutants and birth weight ([54], Table 3). A nested case control study carried out by Rignell-Hydbom and collaborators showed that POPs were not associated with type 1 diabetes [60]. Moreover, we found no published study on the association between in-utero exposures to POPs and type 2 diabetes risks. Inconsistency results across studies can be explained by the following reasons: some studies evaluated the association in background exposure while others used accidental or occupational settings. Moreover, the studies used different sampled sizes and evaluated different POPs. Nevertheless, a risk of type 2 diabetes can be attributed to intrauterine exposure to POPs because of the three way association diabetes, POPs and obesity. Therefore, we can speculate that low birth weight increases the risk of diabetes in adulthood either as a result of under nutrition, through exposure to POPs or both.

**Table 3:** **In-utero exposure to persistent organic pollutants and birth weight**

**Reference**	**Sample size**	**Exposure type**	**Compound used**	**Direction of the association**	**Confounders**
[67]	2246	Occupational	Not stated	No association	Maternal BMI, height, parity, smoking during pregnancy, infant sex
[56]	1322 singleton	Background	CB-153 and p,p’ -DDE	Negative	Maternal age, pre-pregnancy BMI, education, marital status, smoking status, alcohol drinking, parity and newborn’s sex
[55]	286 women	Background	PBDEs	Negative	Maternal age, education, marital status, parity, BMI, country of birth, alcohol and drug use during pregnancy and infant sex
[57]	494	Background	DDT, DDE, HCB and PCBs	Negative	Age, height, pregnancy weight gain, pre-pregnancy BMI, country of origin, residence, parity, education, employment during pregnancy, socioeconomic status
[68]	247 children	Occupational	Not stated	Negative	Maternal smoking, social class and gestational age
[59]	503 women	Background	Not stated	No association	Race, education, age, gestational age at delivery and Child’s sex
[58]	413	Background	PCBs and PBDEs	Positive with PCB and negative with PBDEs	Maternal age, pre-pregnancy BMI, weight gain during pregnancy, education, smoking during pregnancy and sex of the child
[69]	325	Not stated	14 OC pesticides, 7 PCBs and 14 PBDEs	Negative	Age, pre-pregnancy BMI, educational level, and fish consumption

Organochlorine (OC) pesticides, polychlorinated biphenyls (PCBs), polychlorinated dibenzo-p-dioxins (PCDDs), 2,2,4,4,5,5-hexachlorobiphenyl (CB-153), 2,2′-bis(4-chlorophenyl)-1,1-dichloroethylene (p,p′-DDE), di-chlorodiphenyl-trichloro-ethane (DDT), hexachlorobenzene (HCB), body mass index (BMI).

## Conclusion

Type 2 diabetes is increasing at alarming rates in both developed and developing countries. Cross-sectional epidemiological studies have established an association between POPs and type 2 diabetes. Despite different levels of risk in prospective studies and inconsistent results, the causal effect of POPs on diabetes is supported by in-vitro and in-vivo experimental studies. However, detailed mechanistic information on how these pollutants interfere with insulin metabolism is lacking. Furthermore, experimental studies have focused more on the effect of POPs on insulin resistance. Therefore, more research efforts are needed on the interaction of compounds with beta-cell function and/or mass in animal models at human relevant concentrations to further evaluate the hypothesis that environmental pollutants can be additional risk factors for diabetes development. These studies should focus on both peri-natal and in-utero exposure which is thought to affect gene expression through epigenetic mechanisms. Simultaneously epidemiological studies focusing on low level background exposure should be carried out in the general population.

## References

[1] International Diabetes Federation (2013). DIABETES ATLAS.

[2] Carpenters DO (2008). Environmental contaminants as risk factors for developing diabetes. Rev Environ Health.

[3] Arisawa K, Takeda H, Mikasa H (2005). Background exposure to PCDDs/PCDFs/PCBs and its potential health effects: a review of epidemiologic studies. J Med Invest.

[4] Magliano DJ, Loh VHY, Harding JL, Botton J, Shaw JE (2014). Persistent organic pollutants and diabetes: A review of the epidemiological evidence. Diabetes Metab.

[5] Taylor KW, Novak RF, Anderson HA, Birnbaum LS, Blystone C, DeVito M (2013). Evaluation of the Association between Persistent Organic Pollutants (POPs) and Diabetes in Epidemiological Studies: A National Toxicology Program Workshop Review. Environ Health Perspect.

[6] Persky V, Piorkowski J, Turyk M, Freels S, Chatterton R, Dimos J (2012). Polychlorinated biphenyl exposure, diabetes and endogenous hormones: a cross-sectional study in men previously employed at a capacitor manufacturing plant. Environ Health.

[7] Lim JS, Lee DK, David R, Jacobs JR (2008). Association of brominated flame retardants with diabetes and metabolic syndrome in the U.S. Population, 2003–2004. Diabetes Care.

[8] Lee DH, Lee IK, Song KE, Steffes MW, Toscano W, Baker BA (2006). A strong dose–response relation between serum concentrations of persistent organic pollutants and diabetes: results from the National Health and Examination Survey. Diabetes Care.

[9] Codru N, Schymura MJ, Negoita S (2007). Diabetes in relation to serum levels of polychlorinated biphenyls and chlorinated pesticides in adult native Americans. Environ Health Perspect.

[10] Rignell-Hydbom A, Lidfeldt J, Kiviranta H, Rantakokko P, Samsioe G, Agardh CD (2009). Exposure to p, p’9-DDE: A Risk Factor for Type 2 Diabetes. PLoS One.

[11] Lee DH, Steffes MW, Sjödin A, Jones RS, Needham LL, Jacobs DR (2010). Low dose of some persistent organic pollutants predicts type 2 diabetes: a nested case–control study. Environ Health Perspect.

[12] Turyk M, Anderson H, Knobeloch L, Imm P, Persky V (2009). Organochlorine exposure and incidence of diabetes in a cohort of great lakes sport fish consumers. Environ Health Perspect.

[13] Lee DH, Steffes MW, Sjodin A, Jones RS, Needham LL, Jacobs DR (2011). Low dose organochlorine pesticides and polychlorinated biphenyls predict obesity, dyslipidemia, and insulin resistance among people free of diabetes. PLoS One.

[14] Wu H, Bertrand KA, Choi AL, Hu FB, Laden F, Grandjean P (2013). Persistent organic pollutants and type 2 diabetes: A prospective analysis in the Nurses’ health study and meta-analysis. Environ Health Perspect.

[15] Vasiliu O, Cameron L, Gardiner J, Deguire P, Karmaus W (2010). Polybrominated Biphenyls, Polychlorinated Biphenyls, Body Weight, and Incidence of Adult-Onset Diabetes Mellitus. Epidemiology.

[16] Lee DH, Lind PM, Jacobs DR, Salihovic S, van Bavel B, Lind L (2011). Polychlorinated biphenyls and organochlorine pesticides in plasma predict development of type 2 diabetes in the elderly: the prospective investigation of the vasculature in Uppsala Seniors (PIVUS) study. Diabetes Care.

[17] Michalek JE, Pavuk M (2008). Diabetes and Cancer in Veterans of Operation Ranch Hand After Adjustment for Calendar Period, Days of Spraying, and Time Spent in Southeast Asia. J Occup Environ Med.

[18] Silverstone AE, Rosenbaum PF, Weinstock RS, Bartell SM, Foushee HR, Shelton C (2012). Polychlorinated Biphenyl (PCB) exposure and diabetes: results from the Anniston community health survey. Environ Health Perspect.

[19] Ukropec J, Radikova Z, Huckova M, Koska J, Kocan A, Sebokova E (2010). High prevalence of pre-diabetes and diabetes in a population exposed to high levels of an organo-chlorine cocktail. Diabetologia..

[20] Rylander L, Rignell-Hydbom A, Hagmar L (2005). A cross-sectional study of the association between persistent organochlorine pollutants and diabetes. Environ Health..

[21] Cox S, Niskar AS, Narayan KM, Marcus M (2007). Prevalence of self-reported diabetes and exposure to organochlorine pesticides among Mexican Americans: Hispanic health and nutrition examination survey, 1982–1984. Environ Health Perspect..

[22] Lee DH, Lee IK, Jin SH, Steffes M, Jacobs DR (2007). Association between serum concentrations of persistent organic pollutants and insulin resistance among non-diabetic adults: results from the National Health and Nutrition Examination Survey 1999-2002. Diabetes Care..

[23] Rignell-Hydbom A, Rylander L, Hagmar L (2007). Exposure to persistent organochlorine pollutants and type 2 diabetes mellitus. Hum ExpToxicol..

[24] Airaksinen R, Rantakokko P, Eriksson GJ, Blomstedt P, Kajantie E, Kiviranta H (2011). Association between type 2 diabetes and exposure to persistent organic pollutants. Diabetes Care.

[25] Tanaka T, Morita A, Kato M, Hirai T, Mizoue T, Terauchi Y (2011). Congener-specific polychlorinated biphenyls and the prevalence of diabetes in the Saku Control Obesity Program (SCOP). Endocr J..

[26] Uemura H, Arisawa K, Hiyoshi M, Satoh H, Sumiyoshi Y, Morinaga K (2008). Associations of environmental exposure to dioxins with prevalent diabetes among general inhabitants in Japan. Environ Res..

[27] Pesatori AC, Zocchetti C, Guercilena S, Consonni D, Turrini D, Bertazzi PA (1998). Dioxin exposure and non-malignant health effects: a mortality study. Occup Environ Med..

[28] Vena J, Boffetta P, Becher H, Benn T, Bueno-de-Mesquita HB, Coggon D (1998). Exposure to dioxin and non-neoplastic mortality in the expanded IARC international cohort study of phenoxy herbicide andchlorophenol production workers and sprayers. Environ Health Perspect..

[29] Calvert GM, Sweeney MH, Deddens J, Wall DK (1999). Evaluation of diabetes mellitus, serum glucose, and thyroid function among United States workers exposed to 2,3,7,8-tetrachlorodibenzo-p-dioxin. Occup Environ Med..

[30] Wang SL, Tsai PC, Yang CY, Guo YL (2008). Increased risk of diabetes and polychlorinated biphenyls and dioxins: a 24-year follow-up study of the yucheng cohort. Diabetes Care..

[31] Cranmer M, Louie S, Kennedy RH, Kern PA, Fonseca VA (2000). Exposure to 2,3,7,8-tetrachlorodibenzo-p-dioxin (TCDD) is associated with hyper-insulinemia and insulin resistance. ToxicolSci.

[32] Kern AP, Said S, Jackson GW, Michalek EJ (2004). 2004. Insulin Sensitivity following Agent Orange Exposure in Vietnam Veterans with High Blood Levels of 2,3,7,8-Tetrachlorodibenzo-p-Dioxin. J ClinEndocrinolMetab.

[33] Jørgensen ME, Borch JK, Bjerregaard P (2008). A cross-sectional study of the association between persistent organic pollutants and glucose intolerance among Greenland Inuit. Diabetologia..

[34] Færch K, Højlund K, Vind BF (2012). Increased serum concentrations of persistent organic pollutants among prediabetic individuals: Potential role of altered substrate oxidation patterns. J ClinEndocrinolMetab..

[35] DeFronzo RA, Tobin JD, Andres R (1979). Glucose clamp technique: a method for quantifying insulin secretion and resistance. Am J Physiol..

[36] Gauthier MS, Rabasa-Lhoret R, Prud'homme D, Karelis AD, Geng D, Bavel BV (2014). The metabolically healthy but obese phenotype is associated with lower plasma levels of persistent organic pollutants as compared to the metabolically abnormal obese phenotype. J ClinEndocrinolMetab..

[37] Ruzzin J, Petersen R, Meugnier E, Madsen L, Lock EJ, Lillefosse H (2010). Persistent organicpollutantexposureleadstoinsulinresistancesyndrome. EnvironHealthPerspect..

[38] Ibrahim MM, Fjære E, Lock EJ, Frøyland L, Jessen N, Lund S (2012). Metabolic impacts of high dietary exposure to persistent organic pollutants in mice. Toxicol Lett.

[39] Baker AN, Karounos M, English V, Fang J, Wei Y, Stromberg A (2013). Coplanar polychlorinated biphenyls impair glucose homeostasis in lean C57BL/6 mice and mitigate beneficial effects of weight loss on glucose homeostasis in obese mice. Environ Health Perspect..

[40] Hectors TLM, Vanparys C, Van-der-ven K, Martens JA, Jorens PG, Van Gaal LF (2011). Environmental pollutants and type 2 diabetes: a review of mechanisms that can disrupt beta cell function. Diabetologia..

[41] Seefeld MD, Corbett SW, Keesey RE, Peterson RE (1984). Characterization of the wasting syndrome in rats treated with 2,3,7,8-tetrachlorodibenzo-p-dioxin. ToxicolApplPharmacol..

[42] Enan E, Phillip C, Liu C, Matsumura F (1992). 2,3,7,8-tetrachlorodibenzo-p-dioxin causes reduction of glucose transporting activities in the plasma membranes of adipose tissue and pancreas from the guinea Pig. J Biol Chem..

[43] Liu PC, Matsumura F (1995). Differential effects of 2,3,7,8-tetrachlorodibenzo-p-dioxin on the "adipose-type" and "brain-type" glucose transporters in mice. MolPharmacol..

[44] Nishiumi S, Yoshida M, Azuma T, Yoshida K, Ashida H (2010). 2,3,7,8-tetrachlorodibenzo-p-dioxin impairs an insulin signalling pathway through the induction of tumor necrosis factor-a in adipocytes. Toxicol Sci..

[45] Enan E, Liu PCC, Matsumura F (1992). TCDD causes reduction in glucose uptake through glucose transporters on the plasma membranes of the guinea pig adipocyte. J Environ Sci Health..

[46] Leng Y, Karlsson HK, Zierath JR (2004). Insulin signaling defects in type 2 diabetes. Rev EndocrMetabDisord..

[47] Stanley S, Wynne K, McGowan B, Bloom S (2005). Hormonal regulation of food intake. Physiol Rev..

[48] Schug TT, Janesick A, Blumberg B, Heindel JJ (2011). Endocrine disrupting chemicals and disease susceptibility. J Steroid Biochem Mol Biol..

[49] Janesick A, Blumberg B (2011). Minireview: PPAR-gamma as the target of obesogens. Biol: J Steroid Biochem Mol.

[50] Diamanti-Kandarakis E, Bourguignon J-P, Giudice LC (2009). Endocrine-disrupting chemicals: An endocrine society scientific statement. Endocr Rev.

[51] Bastomsky CH (1977). Enhanced thyroxine metabolism and high uptake goiters in rats after a single dose of 2,3,7,8- tetrachlorodibenzo-p-dioxin. Endocrinology..

[52] Zoeller RT, Dowling AL, Vas AA (2000). Developmental exposure to polychlorinated biphenyls exerts thyroid hormone-like effects on the expression of RC3/neurogranin and myelin basic protein messenger ribonucleic acids in the developing rat brain. Endocrinology..

[53] Sala M, Ribas-Fito N, Cardo E, Muga ME, Marco E, Mazon C (2001). Levels of hexachlorobenzene and other organochlorine compounds in cord blood: Exposure across placenta. Chemosphere..

[54] Govarts E, Nieuwenhuijsen M, Schoeters G, Ballester F, Bloemen K, Boer MD (2012). Birth weight and prenatal exposure to polychlorinated biphenyls (PCBs) and dichloro-diphenyldichloroethylene (DDE): A meta-analysis within 12 European birth cohorts. Environ Health Perspect..

[55] Harley KG, Chevrier J, Schall RA, Sjödin A, Bradman A, Eskenazi B (2011). Association of prenatal exposure to PolybrominatedDiphenyl ethers and infant birth weight. Am J Epidemiol.

[56] Wojtyniak BJ, Rabczenko D, Jonsson BA, Zvezday V, Pedersen HS, Rylander L (2010). Association of maternal serum concen-trationsof 2,2, 4,45,5-hexachlorobiphenyl (CB-153) and 1,1-dichloro-2,2-bis (p-chlorophenyl)-ethylene (p, p’-DDE) levels with birth weight, gestational age, and preterm births in Inuit and European populations. Environ Health..

[57] Lopez-Espinosa M-J, Murcia M, Iñiguez C (2011). Prenatal Exposure to Organochlorine Compounds and Birth Size. Pediatrics..

[58] Lignell S, Aune M, Darnerud PO, Hanberg A, Larsson SC, Glynn A (2013). Prenatal exposure to polychlorinated biphenyls (PCBs) and polybrominateddiphenyl ethers (PBDEs) may influence birth weight among infants in a Swedish cohort with background exposure: a cross-sectional study. Environ Health.

[59] Wang L, Wu T, Liu X, Anderson JL, Alamian A, Fu M (2012). Pesticide exposure during pregnancy and low birth weight. South-East Asia Journal of Public Health..

[60] Rignell-Hydbom A, Elfving M, Ivarsson SA, Lindh C, Jönsson BAG, Olofsson P (2010). A nested case–control study of intrauterine exposure to persistent organochlorine pollutants in relation to risk of type 1 diabetes. PLoSOne.

[61] Lee DH, Lee IK, Steffes MW, Jacobs DR (2007). Extended analyses of the association between serum concentrations of persistent organic pollutants and diabetes. Diabetes Care..

[62] Son HK, Kim SA, Kang JH, Chang YS, Park SK, Lee SK (2010). Strong associations between low-dose organochlorine pesticides and type 2 diabetes in Korea. Environ Int..

[63] Gasull M, Pumarega J, Téllez-Plaza M, Castell C, Tresserras R, Lee DH (2012). Blood concentrations of persistent organic pollutants and prediabetes and diabetes in the general population of Catalonia. Environ SciTechnol..

[64] Arrebola JP, Pumarega J, Gasull M, Fernandez MF, Martin-OlmedoP Molina-Molina JM (2013). Adipose tissue concentrations of persistent organic pollutants and prevalence of type 2 diabetes in adults from Southern Spain. Environ Res..

[65] Pal S, Blais JM, Robidoux MA, Haman F, Krümmel E, Seabert TA (2013). The association of type 2 diabetes and insulin resistance/secretion with persistent organic pollutants in two first nations communities in northern Ontario. Diabetes Metab..

[66] Kim KS, Lee YM, Kim SG, Lee IK, Lee HJ, Kim JH (2014). Associations of organochlorine pesticides and polychlorinated biphenyls in visceral vs. subcutaneous adipose tissue with type 2 diabetes and insulin resistance. Chemosphere.

[67] Sathyanarayana S, Basso O, Karr CJ (2010). Maternal Pesticide Use and Birth Weight in the Agricultural Health Study. J Agromedicine..

[68] Wohlfahrt-Veje C, Main KM, Schmid IM, Boas M, Jensen TK, Grandjean P (2011). Lower birth weight and increased body fat at school age in children prenatally exposed to modern pesticides: a prospective study. Environ Health..

[69] Vizcaino E, Grimalt JO, Glomstad B, Fernández-Somoano A, Tardón A (2014). Gestational Weight Gain and Exposure of Newborns to Persistent Organic Pollutants. Environ Health Perspect.

